# Aspen Leaves as a “Chemical Landscape” for Fungal Endophyte Diversity—Effects of Nitrogen Addition

**DOI:** 10.3389/fmicb.2022.846208

**Published:** 2022-03-21

**Authors:** Johanna Witzell, Vicki Huizu Guo Decker, Marta Agostinelli, Carmen Romeralo, Michelle Cleary, Benedicte Riber Albrectsen

**Affiliations:** ^1^Forestry and Wood Technology, Linnaeus University, Växjö, Sweden; ^2^Southern Swedish Forest Research Centre, Swedish University of Agricultural Sciences, Alnarp, Sweden; ^3^Department of Plant Physiology, Umeå Plant Science Center, Umeå University, Umeå, Sweden; ^4^Forest Research Centre (INIA, CSIC), Madrid, Spain

**Keywords:** *Populus tremula*, phenolics, condensed tannins, fungal endophytes, *Chrysomela tremula*, heterogeneity-diversity relationship hypothesis

## Abstract

Abiotic and biotic factors may shape the mycobiome communities in plants directly but also indirectly by modifying the quality of host plants as a substrate. We hypothesized that nitrogen fertilization (N) would determine the quality of aspen (*Populus tremula*) leaves as a substrate for the endophytic fungi, and that by subjecting the plants to N, we could manipulate the concentrations of positive (nutritious) and negative (antifungal) chemicals in leaves, thus changing the internal “chemical landscape” for the fungi. We expected that this would lead to changes in the fungal community composition, in line with the predictions of heterogeneity–diversity relationship and resource availability hypotheses. To test this, we conducted a greenhouse study where aspen plants were subjected to N treatment. The chemical status of the leaves was confirmed using GC/MS (114 metabolites, including amino acids and sugars), LC/MS (11 phenolics), and UV-spectrometry (antifungal condensed tannins, CTs), and the endophytic communities were characterized using culture-dependent sequencing. We found that N treatment reduced foliar concentrations of CT precursor catechin but not that of CTs. Nitrogen treatment also increased the concentrations of the amino acids and reduced the concentration of some sugars. We introduced beetle herbivores (H) as a second treatment but found no rapid changes in chemical traits nor strong effect on the diversity of endophytes induced by herbivores. A few rare fungi were associated with and potentially vectored by the beetle herbivores. Our findings indicate that in a controlled environment, the externally induced changes did not strongly alter endophyte diversity in aspen leaves.

## Importance

Fungal endophytes that colonize plants without causing symptoms may influence the growth and resistance traits of plants. To learn how fungal mycobiomes may potentially support sustainable plant growth, it is important to understand how endophyte communities are regulated by environmental vs. plant inherent factors. In a greenhouse study, we found that the endophyte communities in the leaves of young aspen trees remained rather stable regardless of the subtle changes in chemical quality that were caused by nitrogen fertilization (reduction of potentially antifungal catechins and decrease in sugars and increase in amino acids). A short-term exposure to insect feeding did not cause marked changes to plant chemistry or fungal communities, although some rare fungi occurred only in connection to insect feeding. Thus, in controlled conditions and over a short period of time, the growth environment rather than the internal chemical quality seemed to be a stronger determinant of the fungal diversity in aspen leaves.

## Introduction

Wherever plants grow, in natural ecosystems or cultivated environments, fungal endophytes form a hidden stratum of biodiversity, imbedded inside the plant tissues (Wilson and Lindow, 1994; Bailey et al., 2005; Albrectsen et al., 2010). Currently, studies on such endophyte communities are in the frontline of plant ecology (e.g., Hardoim et al., 2015), and interest in these communities is also boosted by the prospects of utilizing the biosynthetic capacities of the endophytes in plant protection and plant growth promotion in agriculture and forestry (Busby et al., 2016; Witzell and Martín, 2018; Terhonen et al., 2019; Blacutt et al., 2020), and in medical or industrial applications (Nisa et al., 2015; Gouda et al., 2016). Advancement of molecular tools has made it possible to obtain more detailed information about the taxonomic structure of these hidden communities (Jumpponen and Jones, 2009; Albrectsen and Witzell, 2012; Unterseher et al., 2016), but investigations are still challenged by their temporal and spatial dynamics, the complexity of fungal taxonomy, and the lack of knowledge about the roles of fungal species and their inter- and intraspecies interactions inside the plants (Witzell et al., 2014). Especially in the case of large-sized and long-lived forest trees, the mechanisms determining endophyte community composition have remained puzzling (Ragazzi et al., 2003; Albrectsen and Witzell, 2012; Witzell and Martín, 2018).

The ecological and evolutionary processes that shape terrestrial communities include dispersal (movement of organisms across space), drift (stochastic changes in species abundance), speciation (creation of new species), and selection (which involves biotic and abiotic interactions including intra- and interspecific interactions) (Vellend, 2010); moreover, at the landscape level, environmental heterogeneity promotes species richness, as it increases opportunities for niche partitioning (Ben-Hur and Kadmon, 2020). Nutrient availability theory also suggests that plant defenses change character along nutrient gradients, for example, by emphasizing constitutive defenses in nutrient poor soils, to change to investment in both growth and defense in nutrient-richer environments (López-Goldar et al., 2020). In aspen, environmental effects are documented to shape both arthropod community structure (Robinson et al., 2016) and pathogen susceptibility (Bandau et al., 2021).

The impact of soil nutrients on fungal endophyte communities in aspen leaves has received less attention. Trees receive their endophytes mainly from the surrounding environment (horizontal spreading; Clay, 1988; Petrini et al., 1993; Clay and Holah, 1999; Siddique et al., 2022), and the influence of season and climatic conditions on the community structure has been found to be high (Gomes et al., 2018). Edaphoclimatic factors can strongly determine the dispersal and establishment of endophytes in trees (Carroll, 1995; Sieber, 2007), either directly by influencing the quality and quantity of the available inoculum, or indirectly, through effects on the host plant as a substrate for the fungi. Although landing of viable fungal spores on potential host plants is a stochastic event, the chemical environment on and inside the plant may act as selective factor, supporting the germination and growth of some fungi and suppressing others. For instance, several studies have found a connection between endophyte infections and phenolic plant metabolites in trees (Bailey et al., 2005; Albrectsen et al., 2010; Martín et al., 2013; Albrectsen et al., 2018). These ubiquitous plant metabolites are potentially antifungal or fungistatic (Witzell and Martín, 2008), but some fungi may also be able to use them as a carbon source (Blumenstein et al., 2015). Soil fertility is known to influence production of phenolics in plants, and especially the availability of soil nitrogen tends to be negatively correlated with phenolic concentrations in plant tissues (Bandau et al., 2015). Phenolic metabolism is known to readily increase in response to stress, and several phenolics have antioxidant, antimicrobial, or antiherbivore properties (Witzell and Martín, 2008; Gourlay and Constabel, 2019). Because phoretic associations between fungi and insects are likely to be common (Lewinsohn et al., 1994; Rice et al., 2007), insect herbivores may influence the fungal community both directly and indirectly by induction of plant responses. On the other hand, endophytic fungi may also influence the herbivores (Coblentz and Van Bael, 2013), further complicating the interactions.

The goal of our study was to increase the current understanding of how foliar endophyte assemblages in woody plants vary in response to multiple, interacting factors. Our basic hypothesis was that the intrinsic status of phenolic metabolites, dominant defensive chemicals in most boreal and temperate zone trees species, would strongly influence the quality of plants as a substrate for endophytic fungi and thus shape their community structure (Agostinelli et al., 2018; Albrectsen et al., 2018). Moreover, we anticipated that external factors could modify the phenolic status and result in changes in the fungal community. To test this hypothesis, we isolated endophytic fungi from leaves of vegetatively propagated plants of 12 aspen (*Populus tremula* L.) genotypes that were subjected to nitrogen amendment and leaf beetle herbivory, alone and in combination. The genotypes represented a similar chemotype in terms of their content of salicinoid phenolic glycosides (SPGs, low molecular weight phenolics that are characteristic for Salicaceae plants; Keefover-Ring et al., 2014), but differed with regard to concentration of high-molecular weight phenolics, condensed tannins (CTs; Robinson et al., 2012). The plant material thus allowed us to specifically focus on the importance of the latter compounds, which have earlier been identified as potentially antifungal compounds in aspen (Ullah et al., 2017).

We expected that fertilization would generally reduce the concentrations of phenolic metabolites (Witzell and Shevtsova, 2004; Bandau et al., 2015; Decker et al., 2017) in aspen leaves. Furthermore, we expected that it would also reduce the within-group variation in phenolic concentrations, i.e., the phenolic concentration would vary less between N treated replicate plants as compared to the control plant group (Edenius et al., 2012; Albrectsen et al., 2018). We expected that this quantitative response could lead to a more homogenous nutritional niche that would support a less diverse endophytic community than what is found in control plants. In contrast, we hypothesized that herbivory could lead to locally induced accumulation of constitutive or induced phenolics (Sampedro et al., 2011; Massad et al., 2014) or oxidation of phenolics due to tissue damage (Constabel et al., 2000; Salminen and Karonen, 2011). We expected that this would result in a more variable (multidirectional) and compartmentalized chemical environment in leaf endosphere, leading to higher diversity of nutritional niches and thus more diverse fungal communities, as predicted by the heterogeneity–diversity relationship hypothesis (Ben-Hur and Kadmon, 2020). The possible phoretic interactions could add to the diversity of fungal communities in H-treated plants. We used isolation method in order to capture the fast-growing fraction of the total endophyte community because we expected that it would most rapidly respond to the treatment effects in our short-term experiment. To study the metabolic status of the plants, we conducted a global metabolite analysis using GC/MS and completed it with a targeted LC/MS analysis of low-molecular weight phenolics. We also analyzed the concentrations of condensed tannins. The results are discussed within the framework of the heterogeneity–diversity relationship hypothesis (Ben-Hur and Kadmon, 2020).

## Materials and Methods

### Plant Material

Plants belonging to 12 aspen (*P. tremula*) genotypes were chosen from the SwAsp collection (genotypes number 4, 6, 7, 26, 41, 51, 69, 72, 79, 92, 98, and 100; Robinson et al., 2012). The selected clones represented the tremuloides-like chemotype, with four dominating SPGs (salicin, tremuloidin, salicortin, and tremulacin) (Robinson et al., 2012; Keefover-Ring et al., 2014). Aspen plants were produced from *in vitro* tissue cultures (Umeå Plant Science Centre, Umeå, Sweden) and planted into 5-L pots on May 23, 2014. The pots were placed in the greenhouse 20°C, 60% R.H., and a 16:8 h D/N cycle. Lateral branches were removed within the first 4 weeks after planting to promote apical growth.

### Experimental Setup

Two individual plants from each of the 12 genotypes were randomly assigned to one of four treatments: control (C), nitrogen fertilization (N), herbivory (H), and their combination (NH) (*n* = 24 plants per treatment). The whole experiment thus comprised a total of 96 plants. For N treatment, a Weibulls Rika-S^®^ solution (containing 84 gr/liter nitrogen, NH_4_NO_3_) was applied weekly starting from June 17th for 4 weeks. This resulted in a final N input that corresponds to the level of industrial forest fertilization in Sweden, 150 kg N ha^–1^ y^–1^ (Nohrstedt, 2001; Decker et al., 2017). The first fully expanded leaf on each plant was marked on June 17th, and this leaf was later used to assess baseline leaf chemistry (see below).

After 4 weeks of fertilization treatment, a mousseline fabric net was placed over the first three fully expanded leaves that had developed above the marked leaf after the first fertilization event. Five adult aspen leaf beetles (*Chrysomela tremula* Fabricius) were placed into the nets after the last fertilization event and allowed to feed for 5 days (treatments H and NH). The beetles had been reared in the laboratory at Umeå Plant Science Centre since the beginning of May and belonged to the second generation of a culture captured close to Ekebo, in southern Sweden. Empty nets were placed in similar position also on plants in C and N treatments to control for the effect of the mousseline bag.

### Sampling and Growth Measurement

After 5 days of herbivory treatment, the experiment was ended. Leaves were collected from each plant for phytochemical analysis (the leaf below the netted leaves) and for fungal endophyte analysis (the netted leaves). Leaves for the chemical analyses were flash-frozen in liquid nitrogen, freeze-dried, and stored at −20°C until they were ground to fine powder using a bead mill (MM 301 Vibration Mill, Retsch GmbH and Co., KG, Haan, Germany) at 25 Hz for 3 min. The fine powder was stored in vials at −20°C until chemical analyses. Leaves designated for endophyte analysis were collected and stored at 4°C for a maximum of 3 days prior to surface sterilization and endophyte isolation. In case the petiole of a netted leaf was chewed by the beetle (causing wilting or the death of the leaf), those leaves were discarded from further analyses. A total of 90 plants were included in the endophyte analysis. The growth of plants and insects was recorded in the end of the experiment (Supplementary Table 1).

### Phytochemical Analysis

For the global GC/MS analysis, 6.00 (±1.00) mg of leaf powder was extracted in 1 ml methanol:chloroform:water (v:v:v) at 4°C. Deuterated salicylic acid [^2^H_6_] (Isotec, Miamisburg, OH, United States) was used as an internal standard in all samples. Samples were centrifuged at 4°C, and 100 μl of the resulting supernatant was evaporated in vacuum. The residues were resolved in 30 μl of methoxyamine (15 μg/μl in pyridine) and 30 μl of MSTFA. Methyl stearate (30 μl of 15 ng/μl in heptane) was added before analysis, and 1 μl of each aliquot was injected by a CTC Combi Pal autosampler (CTC Analytics AG, Switzerland) into an Agilent 6890 gas chromatograph. The chromatograph was equipped with a 10 m × 0.18 mm fused silica capillary column with a chemically bonded 0.18-μm DB 5-MS UI stationary phase (J&W Scientific, Corston, Bath, United Kingdom). The compound detection was performed in a Pegasus III time-of-flight mass spectrometer, GC/TOFMS (Leco Corp., St Joseph, MI, United States). MATLAB™ R2011b (Mathworks, Natick, MA, United States) was used to quantify the mass by means of integrated peak areas. All pretreatment data procedures, such as baseline correction, chromatogram alignment, data compression, and hierarchical multivariate curve resolution (H-MCR), were performed using custom scripts according to Jonsson et al. (2005). The extracted mass spectra were identified by comparisons of their retention index and mass spectra with libraries of retention time indices and mass spectra (Schauer et al., 2005). Identification of compounds was based on comparison with mass spectra libraries (in-house database) as well as the Kovats retention index. The identified chemicals were quantified by peak area and assigned to metabolite classes (phenolics, amino acids, fatty acids).

For the targeted LC/MS analysis, samples were prepared as above, but the residue was suspended in 25 μl of methanol and 25 μl of 0.1% v/v aqueous formic acid. A 2.0-μl aliquot was injected onto a C18 UPLC™ column (2.1 × 100 mm, 1.7 μm), and chromatographic separation was performed on a LCT Premier TOF/MS in negative mode (Waters, Milford, MA, United States), following the method by Abreu et al. (2011). The standard compounds used in this analysis were from our in-house library (the Swedish Metabolomics Centre: SMC, Umeå, Sweden) including salicinoid standards (salicin, tremulacin, salicortin, and tremuloidin). MassHunter™ Qualitative Analysis software package (version B06.00, Agilent Technologies Inc., Santa Clara, CA, United States) was used to acquire the mass feature extraction (MFE), and extracted features were aligned and matched between samples using Mass Profiler Professional™ 12.5 (Agilent Technologies Inc., Santa Clara, CA, United States). The concentrations of salicinoids were quantified according to the peak area of each compound using linear standard curves. Masses of either or both of the deprotonated ion ([M−H]−) and the formate adduct ([M−H + FA]−) were assessed based on molecular weights according to Abreu et al. (2011) and Keefover-Ring et al. (2014) and guided by retention times where available.

Condensed tannin concentrations were assessed based on a modified Porter’s assay (acid: butanol method) (Porter et al., 1985; Bandau et al., 2015): 10 ± 1 mg fine powder of freeze-dried leaf sample was added to 800 μl of an acetone/ascorbic acid solution (70% acetone, 30% Milli-Q water, with 10 mM ascorbic acid) then incubated with an iron and acid-butanol reagent in boiling water for 1 h. Absorbance was measured on a Spectra Max 190 microplate reader (Molecular Devices, Sunnyvale, CA, United States) at A 550 nm. Procyanidin B2 (C_30_H_26_O_12_, Sigma- Aldrich^®^, St. Louis, MO, United States) was used as the tannin standard.

### Fungal Endophyte Isolation and Identification

Endophytic fungi were isolated following the method described by Albrectsen et al. (2010). From each leaf, 10 segments (about cm^2^) were cut using a sterilized scalpel. The segments were surface-sterilized using 95% ethanol and 2% sodium hypochlorite, and rinsed in sterile water before placing them individually on potato dextrose agar (PDA, Merck 70139-2.5KG) in petri dishes (12 × 12 cm) that had been autoclaved and received added streptomycin to avoid bacterial growth. The leaf segments on the plates were checked for emerging fungal growth every second day, and emerging colonies transferred to new PDA-dishes. Three leaves were harvested from every C and N treated aspen plant; and one to three leaves were harvested from the plants that had been exposed to herbivores. The total number of leaves (and petri dishes) was 228.

The recovered fungal cultures (*n* = 1,642) were classified into 30 distinct morphotypes (MTs) based on their visual morphological traits and growth rate estimated on a four-step scale (fast, medium, slow, very slow). In order to obtain information about the taxonomy of the fungal community, a total of 59 cultures representing the 30 MTs were selected for sequencing. From each culture, plugs of PDA containing growing mycelium were transferred to malt extract broth. After an incubation period of 2 weeks (room temperature, darkness), mycelia were filtered, placed in 50-ml Falcon tubes, and lyophilized for 48 h. Freeze-dried samples were then pulverized in a FastPrep^®^-24 homogenizer (MP biomedicals, Santa Ana, CA, United States). Total genomic DNA was extracted using the E.Z.N.A. SP Plant DNA Kit (Omega Bio-Tek, Inc., Norcross, GA, United States). The extracted nuclear DNA was measured using NanoDrop^®^ ND-1000 (Wilmington, United States). PCR amplification of the internal transcribed spacer (ITS) region of the rDNA was performed with the primers ITS1 and ITS4 (White et al., 1990). Each 50-μl PCR reaction mixture contained 5 μl of 10x PCR buffer, 0.4 μM of each primer, 0.2 mM dNTP, 1.5 mM MgCl_2_, 1U Taq polymerase, and 10 ng fungal DNA. The PCR program consisted of 94°C for 5 min, 33 cycles of 94°C for 30 s, 55°C for 30 s, 72°C for 30 s, and 72°C for 10 min. PCR samples were kept at 4°C before analyzing *via* gel electrophoresis on 1.5% Agarose (BIO-RAD) gel and visualized under UV light with 0.1% GelRed (GelRed™ Nucleic Acid Gel Stain, 10,000X in DMSO). PCR products were purified with the HT ExoSAP-IT High-throughput PCR Product Cleanup (Affymetrix, Santa Clara, CA, United States) following the manufacturer’s instruction. After quantification of DNA concentrations with Qubit fluorometer 3.0 (Life Technologies, Carlsbad, CA, United States), samples were sent to the National Genomics Infrastructure (NGI) at Science for Life Laboratory (SciLifeLab, Uppsala, Sweden). Visualization of raw sequence data was done with the software Chromas (version 2.4.4, Technelysium, South Brisbane, QLD, Australia). All sequences were aligned and manually edited using BioEdit (Ibis Biosciences, Carlsbad, CA, United States). Sequencing data were blasted against best matches in the reference database at NCBI.^1^ The ITS sequence homology for delimiting fungal taxa was set to >98.5% for presumed species (Supplementary Table 2).

### Data Analysis

Kruskal–Wallis non-parametric tests (Table 1) were used to assess the significance of effects on specific chemicals and the summary of the statistical analyses conducted for those relationships that are presented in Figure 2. PCA plots were performed for 114 targeted and non-targeted metabolites from LC/MS and GC/MS and for 11 targeted phenolics (mainly SPGs from LC/MS), respectively, using R version 3.6.1 (2019-07-05) packages: ggplot2,^2^ FactorMineR^3^ (Lê et al., 2008), and factoextra.^4^

**TABLE 1:** Summary of Kruskal–Wallis statistics on treatment effects as presented in Figure 2.

Chemical	Chi-square, df = 3	*p*-value	Significance
Condensed tannins	5.73	0.1255	n.s.
Catechin	25.46	1.239e-05	***
Phenylalanine	31.45	6.81e-07	***
Tryptophan	42.04	3.928e-09	***
Glucose	4.86	0.1817	n.s.
Maltose	21.30	9.122e-05	***

*The non-parametric test was chosen for all comparisons to standardize between compounds with and without normally distributed residuals. The individual chemicals were chosen from the metabolomics data to provide examples of chemical responses to treatment that generally agreed with the compound group: phenolic compounds (as exemplified in condensed tannins and their precursor catechin), the amino acids (phenylalanine and tryptophan), and the sugars (glucose and maltose, with maltose responses varying when compared to the other sugars).*

***P < 0.0001.

Fungal diversity (richness, S; Whittaker, 1972) was tracked as the number of distinct morphotypes (MTs) found in each plant, and the MT abundance (frequency) was recorded as the number of isolates of a given MT per sampled plant (*n* = 24 plants for C and N, and 21 plants for H and NH treatments). Venn diagram was constructed to illustrate the number of shared and unique MTs per treatment (Shade and Handelsman, 2012). Relative abundance was determined for each MT as the number of isolates of the given morphotype divided by the total number of isolates emerged from all samples (*n* = 1642). To examine the possible shifts in the community composition among the different treatments (C, N, H, and NH), a permutational multivariate analysis of variance (Permanova) of relative abundances was performed with the adonis function in vegan package^5^ using 999 permutations, followed by a pairwise comparison with pairwise.adonis (Martinez Arbizu, 2019).

Morphotype richness values were used to create sample-size-based rarefaction (interpolation) and extrapolation (prediction) curves (Colwell et al., 2012; Chao et al., 2014; Hsieh et al., 2016) with an endpoint of 42 individuals and 100 bootstrap repetitions. The curves were generated with the iNEXT (iNterpolation and EXTrapolation) R package^6^ and visualized with ggiNEXT, the ggplot2 extension for iNEXT. To compare the complexity of communities, Simpson’s (*D*) and Shannon (*H*′) diversity indices and Pielou’s index for evenness (*J*′) were calculated using the Vegan package (Oksanen et al., 2016; Supplementary Table 3). The effect of the treatments (C, N, H, NH) on the ecological diversity indexes and on the fungal richness (S) was assessed with an analysis of variance followed by a multiple comparison test (residuals were checked for normality, homoscedasticity, and linearity). When the data did not meet these requirements, a robust ANOVA based on trimmed means was performed with the WRS2 package.^7^ Before all the analyses were performed, data were balanced using a decision tree to generate new samples from the minority classes (H and NH treatments) with an oversampling algorithm (random walk oversampling) from the imbalance package in R^8^ (R Development Core Team, 2016).

## Results

### Treatment Effects on Aspen Chemistry and Growth

**FIGURE 1:**
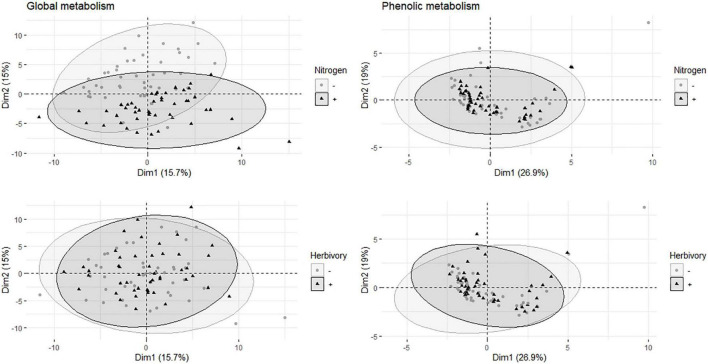
Multivariate analyses of metabolites in the leaves of aspen (*Populus tremula*) plants exposed to environmental endophytic inoculum in controlled, greenhouse conditions: untreated control plants (C) and plants subjected to nitrogen addition (N), herbivory (H), or nitrogen addition and herbivory in combination (NH) (*n* = 24 plants per treatment). Shown are the principal component analysis score plots of 114 targeted and non-targeted foliar metabolites (global analysis, data acquired using gas chromatography/mass spectrometry) and 11 targeted metabolites (the main salicinoid phenolic glycosides and catechin; data acquired using liquid chromatography).

The score plot (PCA) of global metabolite analysis (data included 114 metabolites) showed a weak separation between fertilized and non-fertilized treatments (Figure 1A). The plot of targeted metabolites (11 phenolics, including catechin but excluding condensed tannins) did not show any clear distinction between fertilized and non-fertilized plants (Figure 1B).

**FIGURE 2:**
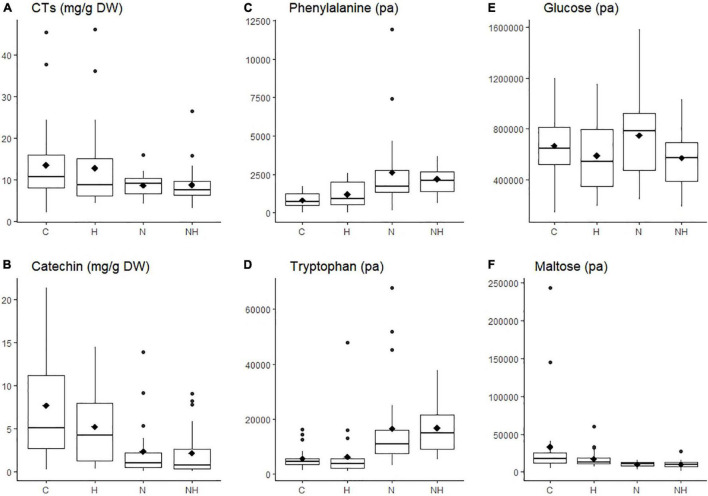
Concentration (mg g^–1^ DW) or level (measured as normalized peak area, pa) of individual chemical compounds in the leaves of aspen (*Populus tremula*) plants exposed to environmental endophytic inoculum in controlled, greenhouse conditions: untreated control plants (C) and plants subjected to nitrogen addition (N), herbivory (H), or nitrogen addition and herbivory in combination (NH) (*n* = 24 plants per treatment). Shown are selected examples for phenolics (**A,B**: concentrations of condensed tannins, CTs, and their precursor catechin), amino acids (**C,D**: phenylalanine and tryptophan), and sugars (**E,F**: glucose, maltose). The data were acquired using liquid chromatography, except for condensed tannins, which we analyzed using a spectroscopy.

Nitrogen treatment reduced the concentration of the CT precursor, catechin, in aspen leaves, and a decreasing (non-significant) trend was observed also in the CT concentration (Table 1 and Figures 2A,B). The lowest within-treatment variation in CTs was found in N-treated plants (Table 1 and Figure 2E), despite the varied genetic background of the trees (12 clones). Overall, the effect of N on chemical characters appeared stronger compared to the effect of H (Table 1 and Figures 2A–F), and there were no significant N × H interactions.

The concentrations of amino acids increased in plants receiving N treatment (Table 1 and Figures 2A,B). Most sugars were somewhat reduced by H and increased by N treatment (data for glucose are shown as an example; Table 1 and Figure 2C). A deviating pattern was found only for maltose, which was reduced by treatments, especially by N, alone and in combination with H (Table 1 and Figure 2D).

Nitrogen treatment had a strong effect on both the above-ground biomass and height of the trees [Supplementary Table 1; ANOVA: *F*_(*df* = 3)_ = 44.07; *p* < 0.0001, *F*_(*df* = 3)_ = 15.96; *p* < 0.0001, respectively], and there was no effect of herbivory (*p* > 0.9).

### Endophyte Diversity in Aspen Leaves

Among the 30 MTs, 18 were identified to class (2 MTs), genus (10 MTs), or species (6 MTs) level (Table 1 and Supplementary Table 2). Of the identified isolates, 17 belonged to *Ascomycota* (class *Dothideomycetes*, *Eurotiomycetes*, *Hypocreales*, and *Sordariomycetes*) and one to *Basidiomycota* (MT25: *Rhodotorula* sp.). The three most common morphotypes across the treatments, *Ramularia* sp. (MT1), and the unidentified morphotypes MT2 and MT3 had 576 isolates (35% of all isolates), 336 (20% of all isolates), and 329 (20% of all isolates), respectively. A total of 18 MTs were captured as a maximum of three isolates (<0.2% relative abundance) and were thus considered as rare MTs.

**FIGURE 3:**
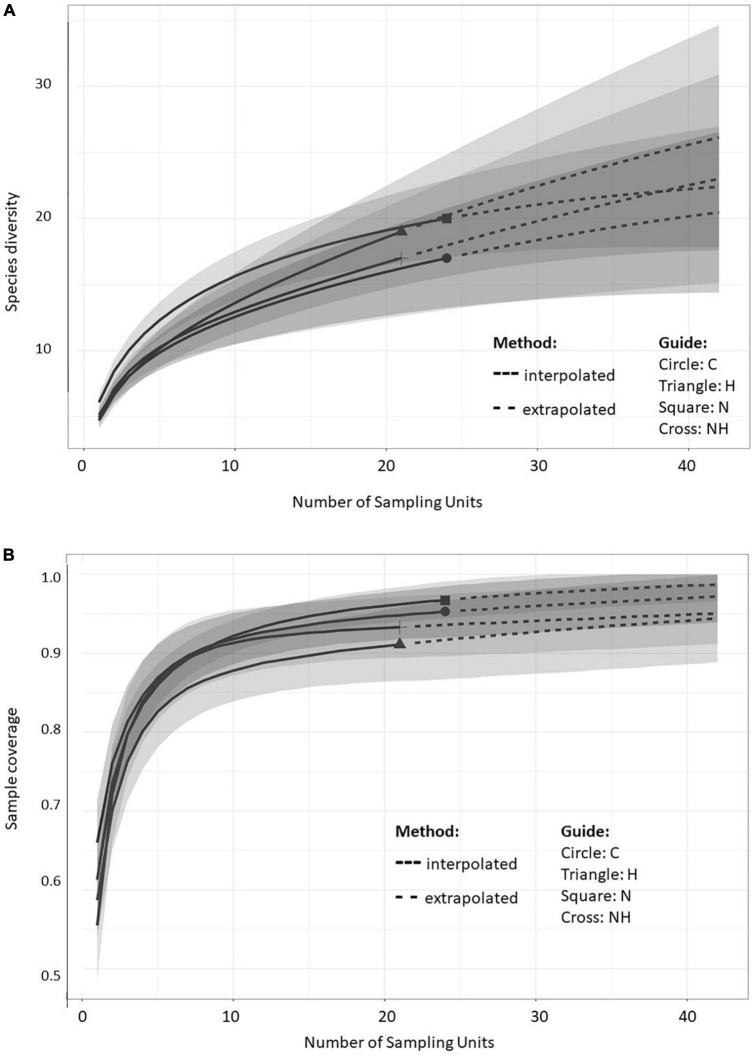
Sample-size-based **(A)** and coverage-based **(B)** rarefaction (solid line) and extrapolation (dotted line) curves comparing fungal morphotype richness in the leaves of greenhouse-cultivated aspen (*Populus tremula*) plants (*n* = 96): untreated control plants (C) and plants subjected to nitrogen addition (N), herbivory (H), or nitrogen addition and herbivory in combination (NH) (*n* = 24 plants per treatment). The shaded areas represent the 95% confidence intervals. The different symbols represent the reference samples.

The sample size-based rarefaction and extrapolation curves (Figure 3A) are predicted to accurately estimate the number of MTs per sampling unit (tree) within the double number of species when compared to the reference sample size (Chao et al., 2014). At least two fungal isolates were recovered from each plant, and the highest increase in diversity was obtained when increasing the number of sampled trees from 1 to 5 within all the treatments. The non-asymptotic rarefaction curves suggest that additional sampling efforts would yield more MTs, especially in H treatment. However, the coverage-based rarefaction and extrapolation curves (Figure 3B) suggested that the sample size (24 trees for C and N each, and 21 for H and NH) allowed high completeness with the method that was used: the sample coverage values were 0.95, 0.97, 0.91, and 0.93 for C, N, H, and NH, respectively.

### Treatment Effects on Endophytes

The treatments caused significant differences in endophyte richness (robust ANOVA, *F* = 3.70, *p* = 0.02) and relative abundance (Permanova, *F* = 1.67, *p* = 0.04). These differences were located between N and H treatments (*p* = 0.01 for richness; and *F* = 2.92, *p* = 0.02, for abundance), while the rest of combinations did not result in significant differences. Together, the isolates belonging to the three dominating MTs made up 81% of all isolates in C plants, but their proportion tended to decrease by treatments: in N and H treatments, they made up 73% of all isolates, and in NH 71%. At the same time, the relative abundance of the other (less frequent, and rare) morphotypes increased correspondingly (Table 1).

The Shannon index (H’) did not differ among the treatments (ANOVA, *F* = 1.54, *p* = 0.21), with mean (±se) and SE values 1.35 (±0.07) for C; 1.50 (±0.06) for N, 1.24 (±0.10) for H, and 1.36 (±0.08) for NH. Likewise, the Simpson index (D) values did not vary among treatments (0.68 ± 0.02 for C; 0.72 ± 0.02 for N; 0.62 ± 0.04 for H; and 0.68 ± 0.03 for NH; robust ANOVA, *F* = 0.41, *p* = 0.74) nor did the values of Pielou’s measure of evenness (J) (0.85 ± 0.02 for C; 0.84 ± 0.02 for N, 0.82 ± 0.03 for H and 0.86 ± 0.02 for NH; robust ANOVA, *F* = 1.03, *p* = 0.39).

**FIGURE 4:**
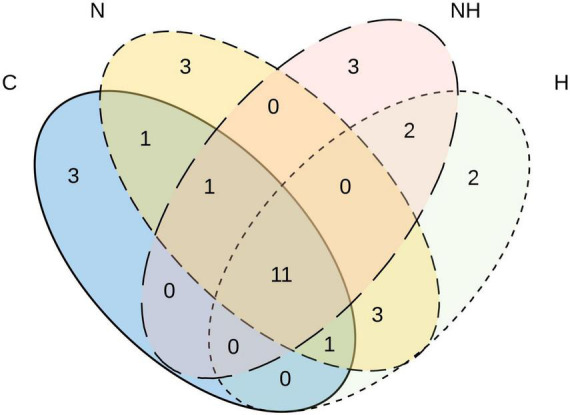
Venn diagram showing the number of unique and shared morphotypes of fungal endophytes in the leaves of greenhouse-cultivated aspen (*Populus tremula*) plants untreated control plants (C) and plants subjected to nitrogen addition (N), herbivory (H), or nitrogen addition and herbivory in combination (NH) (*n* = 24 plants per treatment).

In total, 11 MTs were shared among all four treatments (Figure 4), of which six morphotypes were identified at least to the genus level (*Ramularia* sp., *Cladosporium* sp., *Penicillium olssonii*, *Arthrinium* sp. and 2, *Penicillium* sp.) and five remained unknown (Table 2). Three rare MTs were recovered only from C plants, including a Dothideomycetes and a *Coleosporium* species, and three others from N-treated plants (a Dothideomycetes, *Physalospora scripi*, and an unknown species). Three rare MTs, including the identified *Aureobasidium* species (*A. microstictum* and *A. pullulans*), were found only in H treatments (Table 2).

**TABLE 2:** An overview of morphotype identity and abundance (calculated as the number of isolates of a morphotype per plant normalized after the total number isolates) from leaves of aspen clones belonging to the Swedish Aspen collection (Luquez et al., 2008) and divided after treatment (C, control; N, nitrogen fertilization; and subsequent herbivory H of controls and nitrogen treated plants, H and NH, respectively).

Morphotype	Identity	Treatment			
		
		C	N	H	NH
Total nr of isolates		543	520	268	311
MT1	*Ramularia* sp.	8.83	6.88	5.14	4.33
MT2		4.71	5.13	1.48	3.29
MT5		4.83	3.92	2.71	2.95
MT13	*Cladosporium* sp. 1	1.08	1.29	0.71	0.76
MT18		0.88	0.83	0.38	0.43
MT12	*Penicillium olssonii*	0.42	0.67	0.57	0.81
MT22	*Arthrinium* sp. 1	0.54	0.75	0.52	0.52
MT15	*Penicillium* 1	0.54	0.54	0.10	0.52
MT26		0.29	0.29	0.43	0.71
MT17	*Penicillium brevicompactum*	0.00	0.46	0.19	0.00
MT23	*Arthrinium* sp. 2	0.08	0.25	0.05	0.05
MT3	*Fusarium oxysporum*	0.04	0.04	0.00	0.05
MT4		0.00	0.04	0.05	0.00
MT6		0.17	0.08	0.05	0.05
MT7		0.04	0.00	0.00	0.00
MT8	*Dothideomycetes* 1	0.00	0.08	0.00	0.00
MT9	*Dothideomycetes* 2	0.04	0.00	0.00	0.00
MT10		0.00	0.00	0.05	0.05
MT11		0.00	0.00	0.05	0.00
MT14	*Cladosporium* sp. 2	0.04	0.00	0.00	0.00
MT16		0.00	0.04	0.00	0.00
MT19		0.04	0.04	0.05	0.00
MT20	*Penicillium* sp. 2	0.00	0.00	0.00	0.05
MT21	*Penicilium* sp. 3	0.04	0.08	0.00	0.00
MT24	*Arthrinium* sp. 3	0.00	0.00	0.05	0.00
MT25	*Rhodotorula* sp.	0.00	0.21	0.14	0.00
MT27	*Aureobasidium microstictum*	0.00	0.00	0.00	0.14
MT28	*Aureobasidium pullulans*	0.00	0.00	0.05	0.05
MT29		0.00	0.00	0.00	0.05
MT30	*Physalospora scirpi*	0.00	0.04	0.00	0.00

## Discussion

Nitrogen treatment induced some of the expected changes in the quality of aspen leaves: in particular, the global metabolite analysis and catechin levels highlighted the difference in the chemical profile between fertilized and non-fertilized aspen plants. A characteristic change in the global metabolite pool seemed to be due to increased concentrations of amino acids and a decrease in some sugars, which could reflect the expected positive effect of N on growth and associated metabolic changes in the plants (Supplementary Table 1, growth data). While the N effect on CTs was not significant, we found lower concentration of CTs in the leaves of fertilized plants, and the N treatment also tended to reduce the variation in CT concentration among the studied trees. Thus, nitrogen fertilization seemed to render the leaves to a more nutritious and less toxic environment, assuming bioactive effects of catechins (Gaur et al., under review), but also a more homogenous substrate for the fungi.

In contrast to our initial expectation, however, the detected quantitative changes were not accompanied by a more uniform fungal community in aspen leaves when compared with the control plants. Thus, our findings did not support for the classic heterogeneity–diversity relationship hypothesis among the endophyte community in aspen leaves (i.e., the apparently more homogenous chemical environment, especially in terms of condensed tannins, was not accompanied by a lower endophyte diversity). However, recent studies suggest that heterogeneity–diversity relationships may be non-linear and more complex than expected from the niche-based perspective (Ben-Hur and Kadmon, 2020). Resource availability may further alter the relative investment in different defense mechanisms in plants (López-Goldar et al., 2020), and earlier studies have shown that growing environment also determines aspens’ investment in CTs (Decker et al., 2017). In our greenhouse study, the CT level was generally low as compared to other studies where the detection has been done against the same commercial standard (e.g., Bandau et al., 2016), but it is unclear if the low CT level had consequences for the responses of the fungal community in our study. A deeper taxonomic analysis, using culture-independent approaches, may be needed to reveal these relationships. Clearly, more detailed information about nutritional niches and functional dynamics of different endophytic fungi (Blumenstein et al., 2015; Paungfoo-Lonhienne et al., 2015) would facilitate the analyses of the niche-dependent mechanisms behind the observed patterns in fungal diversity.

We expected that leaf herbivory would increase both the compositional (magnitude) and configurational (spatial) chemical heterogeneity in the leaf tissues by inducing changes in phenolic metabolism, and that this change would lead to a more complex community structure among the relatively fast growing, culturable fraction of endophyte communities. However, we did not find evidence for the expected changes in CTs or other phenolics in response to herbivory. Possibly, the impact of herbivory could have been stronger in younger leaves (a systemic induction) or after a longer feeding period. Moreover, the MT richness in H-treated plants did not increase as compared to C plants. Interestingly, we found that three of the MTs were found only in connection to herbivory, supporting the view that insects may influence the fungal diversity by carrying specific fungi to the plants (Albrectsen et al., 2018). Wounding by herbivores may also open up more entry points for the ubiquitous fungi such as *Aureobasidium pullulans*, which was identified among the three H-treatment-specific MTs and is commonly found as an epiphyte and endophyte in different environments (Martín et al., 2013; Albrectsen et al., 2018).

The combined effect of N and H on the chemical traits or fungal diversity seemed to be dominated by the N effect. The longer impact time of N was likely to accentuate its effect as a stronger bottom-up force and allowed it to shape the quality of leaves as a substrate for fungi more than the short-term herbivory. Fungi transmitted to plants by the feeding beetles would probably need a longer incubation time than what was possible in our study before they can be captured using the culturing approach. It should also be noted that in our study, the insects were reared in laboratory conditions and thus exposed to only a limited environmental inoculum. Therefore, the results of this study may underestimate the importance of insect-mediated facilitation of infections that occurs in natural environments.

In accordance with the universal pattern of species abundance distribution (McGill et al., 2007), we found that the culturable endophyte community in aspen leaves was composed of a few common species (the main community member *Ramularia* spp. and two unknown ones) but many rare species. The identified taxa represented *Ascomycota* commonly found as endophytes in trees (Arnold et al., 2007; Rodriguez et al., 2009; Unterseher, 2011). The only *Basidiomycota* species identified, *Rhodotorula* sp., has been reported as a common endophyte in trees in previous studies (Jumpponen and Jones, 2010; Unterseher, 2011; Rajala et al., 2014), and it has also been linked to herbivory treatment (Albrectsen et al., 2018). In our study, however, only weak link was found between *Rhodotorula* sp. and herbivory. Intriguing was also that some common tree endophytes, such as *Alternaria* and *Phomopsis* (Albrectsen et al., 2010; Crous and Groenewald, 2013; Martín et al., 2013), were not detected, possibly because the greenhouse acted as a filter for the environmental inocula and reduced the frequency of otherwise common endophytes. Moreover, the time point of sampling in early summer may be reflected in the community composition: the species diversity is likely to increase during the season as the infections accumulate. Among the other genera identified, *Cladosporium*, *Ramularia* (anamorph *Mycosphaerella*), and *Physalospora* have been previously described as plant pathogens (Thomma et al., 2005; Tadych et al., 2012; Videira et al., 2016), but no signs of pathogen attacks were found on the leaves, suggesting that the conditions in our experiment supported endophytic lifestyle of these and other fungi.

In conclusion, our results indicate that the culturable fraction of fungal endophyte community in aspen leaves is rather stable against the directional alterations induced in the chemical “landscape” within the leaves by nitrogen fertilization or disturbances due to herbivory. This is in line with the results of the study by Christian et al. (2016) who reported high resistance among endophyte communities against biotic and anthropogenic perturbations. However, more research is needed to better comprehend the nutritional requirements of endophytes and the signals influencing the dynamics of the microbial communities in the endosphere. This is particularly important in future conditions where climate change may directly or indirectly cause major alterations in nutrient availability for plants (Kreuzwieser and Gessler, 2010). While our results did not agree with the expectations we made based on the heterogeneity–diversity relationship hypothesis, we propose that by implementing theoretical frameworks of community ecology, it is possible to gain new insights into the processes and traits driving the structure and functions of endophyte communities in perennial plants, such as forest trees.

## Data Availability Statement

The data are available at doi: 10.5061/dryad.3j9kd51kp after publishing (https://datadryad.org/stash/share/USovDaEtgAairebD6HyROVNXuDkv0V8PsQjtp-0-AFY).

## Author Contributions

VD and MA planned the research supervised by BA and JW. VD performed the experiments and performed the chemical analyses. MA, CR, BA, and MC performed data analyses. VD and MA wrote the first draft. JW and BA wrote the manuscript. All authors contributed to the final version of the manuscript.

## Conflict of Interest

The authors declare that the research was conducted in the absence of any commercial or financial relationships that could be construed as a potential conflict of interest.

## Publisher’s Note

All claims expressed in this article are solely those of the authors and do not necessarily represent those of their affiliated organizations, or those of the publisher, the editors and the reviewers. Any product that may be evaluated in this article, or claim that may be made by its manufacturer, is not guaranteed or endorsed by the publisher.

## Footnotes

1
http://www.ncbi.nlm.nih.gov/

2
https://cran.r-project.org/web/packages/ggplot2/

3
https://cran.r-project.org/web/packages/FactoMineR/index.html

4
https://cran.r-project.org/web/packages/factoextra/index.html

5
https://cran.r-project.org/web/packages/vegan/index.html

6
https://cran.r-project.org/web/packages/iNEXT/index.html

7
https://cran.r-project.org/web/packages/WRS2/index.html

8
https://cran.r-project.org/web/packages/imbalance/index.html
